# An Innovative Approach to Educating Medical Students About Personal Finance

**DOI:** 10.7759/cureus.15579

**Published:** 2021-06-10

**Authors:** Kabir Grewal, Michael J Sweeney

**Affiliations:** 1 Department of Clinical Sciences, Florida State University College of Medicine, Tallahassee, USA; 2 Department of Surgery, Florida State University College of Medicine, Tallahassee, USA

**Keywords:** personal finance, medical school debt, physician loans

## Abstract

Background

The lack of financial literacy among medical students and physicians is a well-documented problem that is yet to be sufficiently addressed. The full immersion of physicians into their scientific education and medical training makes it challenging to also comprehensively learn about personal finance. With the vast debt that most medical students undergo, it is crucial to focus on this issue.

Methodology

To address this challenge, faculty at The Florida State University College of Medicine (FSU COM) have created a fully online personal finance elective course for fourth-year medical students. This course, titled *Personal Finance for the New Physician*, focuses on critical fiscal topics that are directly relevant to future doctors. The elective is delivered remotely through the utilization of online discussion boards, weekly video conferences, and recorded lectures by financial experts. This innovative distribution model ensures that busy students can educate themselves regardless of time or location.

Results

Initially offered in January of 2019, *Personal Finance for the New Physician* quickly became the most chosen elective at FSU COM. To determine the course’s efficacy, students completed a questionnaire at both the start and end of the elective. The results show substantial improvements in fiscal knowledge and confidence in managing personal finances. The average financial competence score of those who completed the course increased from a 2.25/5 to a 4.45/5. Feedback from students so far has been exceedingly positive.

Conclusions

*Personal Finance for the New Physician* has been effective at addressing the lack of financial knowledge that is prevalent among medical students. Additionally, the unique online nature of this course would allow for realistic expansion to other medical programs. Course directors will continue to receive feedback from students, financial experts, and physicians to modify and improve the elective.

## Introduction

During medical school, students spend countless hours learning about the scientific intricacies of the human body. Through clinical training, students learn how to properly care for patients and become the best physicians possible. This process of total immersion into the art and science of medicine often prevents students from focusing on other topics that are important to their career and life. One such critical area is personal finance. A lack of financial knowledge and planning among physicians can lead to an uncertain future burdened by poor decision-making. This dearth of knowledge often first becomes an issue during college and medical school when many students do not understand the full consequences of student debt. As a result, we believe this lack of knowledge should be adequately addressed in medical school, specifically during the fourth year. This is the time period during which curriculum time is available and student interest in personal finance increases in anticipation of employment. However, the fourth year is also when students tend to travel for residency interviews and engage in external clinical rotations. These factors demand an innovative approach for the delivery of education content that offers both temporal and geographical flexibility. This inspired us to create a personal finance education elective consisting of recorded lectures by content experts and weekly live video discussions.

Currently, the average total education debt faced by a medical school graduate surpasses $251,600, and over the standard course of four years of premedical studies, four years of medical school, and three to seven years spent in residency training, doctors are often forced to allow significant expansion of this debt load prior to implementing a repayment strategy [1]. The current trend of increasing medical sub-specialization only exacerbates this challenge for new physicians [2]. The ever-expanding regulatory environment and decreasing reimbursements are but some of the additional reasons financial education will become more important than ever for doctors [2]. As a result, it seems essential for physicians, including those in training, to be adequately prepared to manage their debt effectively within the context of their overall personal finances.

Major concerns moving forward include the detrimental emotional and psychological impacts that a lack of financial knowledge can have on physicians, especially those early in their professional careers. The transition from the protected medical school environment, to the well-structured world of residency, to independent or employed practice can be confusing and intimidating. These transitional difficulties are often exacerbated by feelings of inadequate preparation, unprecedented responsibilities, and the struggle to adjust to one’s role in a hierarchical healthcare system. A recent survey regarding this topic found that as many as 82% of medical interns reported symptoms of burnout [3]. A less anticipated result showed that, of the challenges frequently faced by interns, financial worries ranked the highest, with nearly 72% of interns reporting high or very high stress from this concern [3]. Many new physicians use words such as “frightening” and “terrifying” to describe purchasing insurance or dealing with their student debt [4]. A separate survey found that even among program directors, the presence of considerable financial stress was significantly correlated with higher levels of emotional exhaustion and depersonalization [5]. We believe that providing earlier financial training can help alleviate these effects, and that reducing external stress on physicians may improve overall wellness, professional satisfaction, as well as patient interactions and outcomes.

The typical premedical student is required to take dozens of rigorous science courses while gaining clinical exposure as well as studying for the Medical College Admission Test. Once in medical school, these students must then fully immerse themselves into their medical studies and clinical rotations. Due to a lack of time, interest, or awareness, many of these students fail to recognize personal finance as important to their future until they near the end of their medical education. This can be seen at the Florida State University College of Medicine (FSU COM), where, historically, less than 5% of medical students have significant formal education in personal finance, business principles, or any related disciplines. A broader survey completed by over 1,000 medical students from various medical schools found that only 56.6% of M4s received personal finance counseling or education during medical school [6]. Respondents to this same survey also scored an average of only 47.7% correct on a basic financial literacy quiz [6]. These data results provide evidence of the need to improve the financial education of medical students.

## Materials and methods

Addressing the issue

Over the last few years, multiple initiatives have been made across the country to alleviate this problem. For example, the number of joint MD/MBA programs in the United States has risen from six to nearly seventy within the last 25 years [7]. Although this option delivers general business knowledge and skills, these programs do not focus on personal financial management and are not a fully viable solution. Most medical students are unlikely to spend one to two years and undergo additional debt to pursue yet another degree. A preferable solution should instead require less interruption of basic medical education and be more readily available to a wider population of students while not adding debt [8].

As a result, several institutions have begun experimenting with different ways to educate students about finance within the confines of the traditional four-year medical school curriculum. In 2013, students at the Michigan State University College of Human Medicine created an elective course to learn about the basics of personal finance, student debt handling, and business management [9]. Nearly 85% of students felt that they benefited from taking the elective, and 30% believed that the *Medical Business and Finance* elective should become mandatory in their medical school curriculum [9]. A similar initiative was made at the University of Arkansas for Medical Sciences, where a 10-week *Business of Medicine* elective course has been held annually. Data reported from 2015-2018 show that the percentage of fourth-year medical students who chose to enroll in this course increased from 43% to 77% [10]. Of those who took the course, 98.9% said that the knowledge gained in this course would be helpful in the future [10]. These sorts of positive results have encouraged us at the FSU COM to create our own personal finance curriculum and to develop an innovative delivery model to ensure its availability to all our medical students.

The FSU COM approach

The FSU COM utilizes a distributive model of clinical education and training in which students spend their final two years of medical school at one of six regional campuses and three additional sites across the state of Florida. While this model has proven effective at preparing graduates for residency and beyond, it posed certain challenges to designing and delivering a personal finance elective to fourth-year medical students. Most notably, we needed to create a course that was easily accessible across multiple locations. Through the input and contributions of various faculty members at the FSU College of Business, we developed a curriculum that allowed for the combination of recorded video lectures, supplementary readings on relevant topics, and weekly live video conferences between students and course directors. The implementation of video technology and recordings enabled students to access the learning modules at any time from anywhere. This led to near-universal accessibility for participants even while juggling residency interviews, clinical rotations, and unanticipated events such as the coronavirus disease 2019 lockdown.

Personal Finance for the New Physician

The course, titled *Personal Finance for the New Physician*, is delivered over four weeks and encompasses nine different topics that are directly applicable to future physicians (see Table 1). These relevant subjects include debt management, risk insurance, retirement planning, and contract negotiations. Recorded video lectures on each topic are created by FSU College of Business professors and additional content experts with graduate-level expertise in their fields. To protect the integrity of the course, we at FSU deemed it crucial that the contributing faculty had no financial conflicts of interest associated with any of the material they presented. This ensures the information taught to medical students is as unbiased as possible. Upon watching the lectures and reading the required materials, participants are required to participate in the online discussion sessions. At the conclusion of the course, students are required to submit a reflection project detailing their own personal financial strategy moving forward.

**Table 1:** Course syllabus outline for Personal Finance for the New Physician.

Topic	Description
Practice Models and Contract Negotiation	Structural Options, Practice Metrics, Employment Contracts
Risk Management and Insurance	Life, Disability, Home, and Auto Insurance, Business Insurance, General and Professional Liability
Financial Principles	Time Value of Money, Compound Interest, Wealth Generation
Financial Planning	Components of a Comprehensive Plan, Planner Selection, Tax Strategies, Asset Allocation, Financial Advisors
Savings and Investments	Asset Classes, Securities, Investment Vehicles, Portfolio Management, Asset Protection
Debt Management	Student Loans, Banking, Real Estate, Consumer Loans
Retirement Planning	Informational Websites, Deferred Compensation, Employer Matching, Asset Development
Qualified Retirement Plans	IRA options, Roth vs Traditional, 401(k), 403(b), etc.
Estate Planning	Wills and Trusts, Beneficiaries, Tax Implications, College Savings, Charitable Contributions

## Results

A short questionnaire was given to students both prior to and after completing the elective. This 10-question survey asked students to self-assess their knowledge about various fiscal topics. Blinded survey responses were anonymous, participation was voluntary, and no privileged, confidential, or personal identifying information was included. The survey design provided an Institution Review Board exemption by the research committee at FSU.

Of the 201 students who have taken the course thus far, 160 students answered the initial questionnaire and 123 completed the follow-up survey upon completion of the course. After assigning a numerical value to each answer, we calculated a mean competence score for each topic. The results indicated a significant increase across the board in student confidence in fiscal knowledge and in facing personal financial issues. Substantial increases were observed in responses to survey questions on each of the covered topics, as shown in Table 2, with the overall self-assessed mean competence score rising from 2.25/5 to 4.45/5.

**Table 2:** Pre-/Post-course student survey results (2019-2020). Students answered “Strongly Disagree” (1), “Disagree” (2), “Neutral” (3), “Agree” (4), or “Strongly Agree” (5) to each question. The corresponding numerical values were used in calculating the average competence scores. Out of the 160 students who filled out the pre-course survey, 123 also filled out the post-course survey.

Survey questions	Average competence score (1-5)	
	Pre-course (n = 160)	Post-course (n = 123)
Question 1: I feel confident about my personal finance knowledge and skills	2.28	4.29
Question 2: I am comfortable managing my budget and finances	2.98	4.46
Question 3: I am aware of various structural options for medical practice along with the general advantages and disadvantages of each	2.16	4.37
Question 4: I am able to assess my current and future need for risk management and evaluate various insurance options from a personal and professional perspective	1.89	4.44
Question 5: I understand basic financial principles such as time value of money and wealth accumulation	2.93	4.62
Question 6: I am familiar with the basics of tax planning, asset allocation, and financial advisor selection	1.95	4.41
Question 7: I can distinguish between savings and investments and can describe the basic characteristics of various asset classes	2.52	4.57
Question 8: I appreciate the importance of debt management in personal finance and can build a strategy to address the issue	3.08	4.66
Question 9: I can discuss the importance of long-term planning for future financial obligations, including retirement, college education costs, charitable giving, and estate planning	2.86	4.64
Question 10: I am aware of various options for retirement planning and can discuss the basics and tax implications of each	2.01	4.38

## Discussion

This course was first offered in January of 2019 and quickly became the most chosen elective among fourth-year medical students at FSU COM. This highlights the demand and desire among medical students to improve their personal finance knowledge. The preliminary results from those who have taken *Personal Finance for the New Physician* are encouraging and showcase the efficacy of the elective. The survey responses also help exhibit the effectiveness of the course. In their Final Project submissions, most students expressed gratitude for the opportunity to participate in this elective. Satisfaction with the scope, structure, and pedagogy of the course has also been extremely high. The fully virtual nature of this course could soon help address the widespread deficiency of personal finance knowledge among medical students in this country.

## Conclusions

To educate our fourth-year medical students about personal finance, the FSU COM has designed and implemented an innovative course to provide accessibility to students regardless of their educational site or individual schedules. Initial results from our elective course indicate that it has been very successful in achieving the objective of better preparing our graduates to manage their personal finances. Those who have completed the course report dramatic increases in their financial knowledge as well as enhanced confidence in their ability to effectively manage future fiscal challenges. As a result, we believe that *Personal Finance for the New Physician* has and will continue to help ensure that our graduates receive a truly comprehensive education prior to transitioning to residency and on to their professional careers. The course’s online design provides distributive access, flexible content engagement, and offers scalability which can be expanded or replicated in response to increased demand. Therefore, this sort of course structure could yield itself to future widespread adoption by medical students across the country.

Moving forward, we will continue to gather feedback from students to best improve and enhance the course’s learning modules and overall structure. We will also acknowledge suggestions from students about which fiscal topics we should further emphasize. Furthermore, we will pursue suggestions and critiques from both practicing physicians and financial experts regarding how to best advance the elective.
